# Supplementary material for: STT3-dependent PD-L1 accumulation on cancer stem cells promotes immune evasion
Source: Nat Commun. 2018 May 15;9:1908. doi: 10.1038/s41467-018-04313-6 (PMC5954021; doi:10.1038/s41467-018-04313-6)
Supplement: Supplementary file 1 — Supplementary Information [file 41467_2018_4313_MOESM1_ESM.pdf]

Supplementary Information

STT3-dependent PD-L1 accumulation on cancer stem cells promotes immune evasion

Hsu et al.

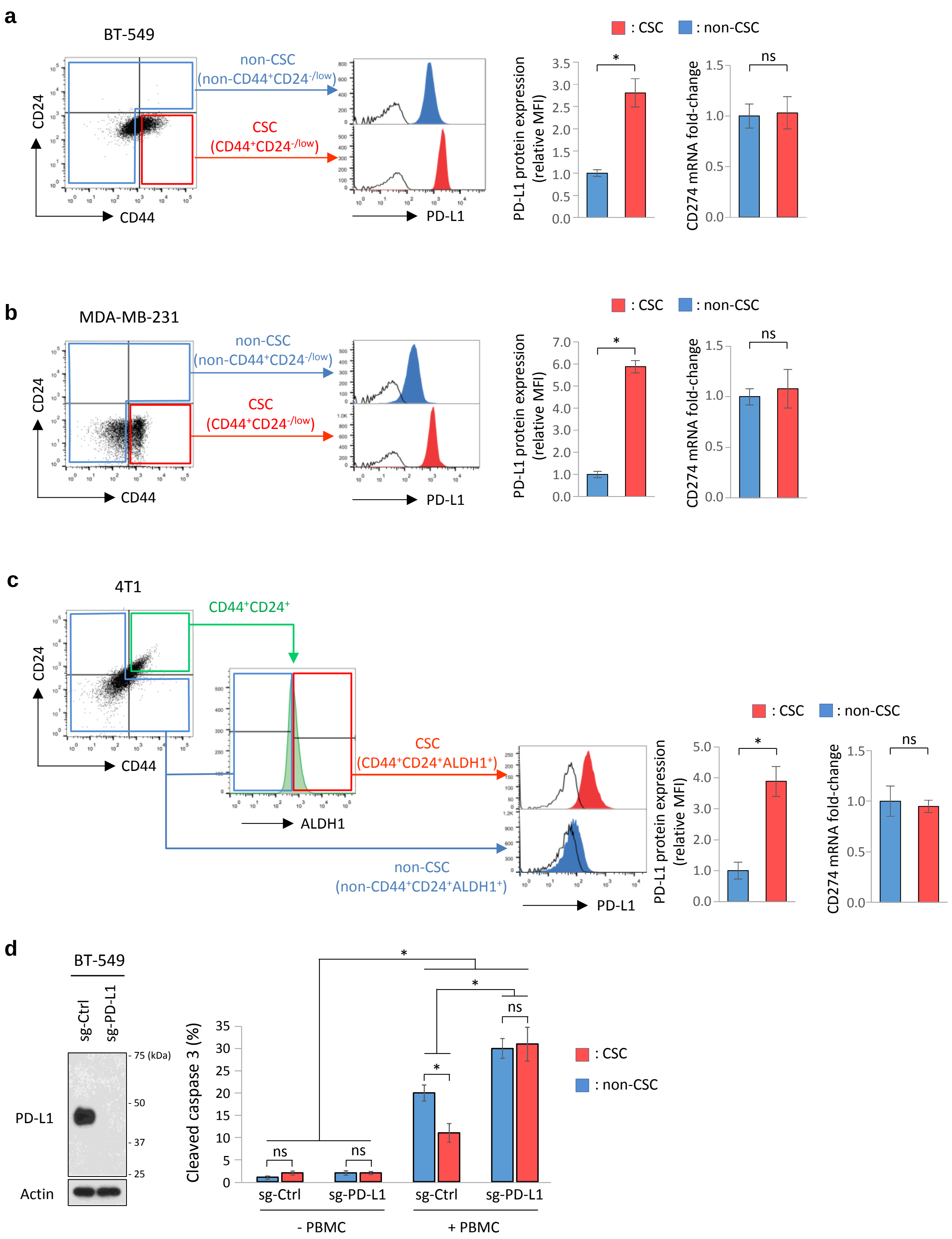
a
BT-549
non-CSC
(non-CD44+CD24-/low)
CSC
(CD44+CD24-/low)
CD24
CD44
PD-L1
: CSC
: non-CSC
PD-L1 protein expression (relative MFI)
CD274 mRNA fold-change
ns
b
MDA-MB-231
c
4T1
CD44+CD24+
ALDH1
CSC
(CD44+CD24+ALDH1+)
non-CSC
(non-CD44+CD24+ALDH1+)
d
BT-549
sg-Ctrl
sg-PD-L1
PD-L1
Actin
75 (kDa)
50
37
25
Cleaved caspase 3 (%)
- PBMC
+ PBMC

Supplementary Figure 1. Compared with non-CSC populations, CSC populations have higher PD-L1 expression, which contributes to CSC immune evasion.

(a, b, c) Flow cytometric analysis of PD-L1 protein expression and qRT-PCR analysis comparing PD-L1 (CD274) mRNA levels between the CSC (red gate) and non-CSC (blue gate) populations of TNBC cells, BT-549 (**a**), MDA-MB-231 (**b**) and 4T1 (**c**). The CSC populations of human TNBC cells (BT-549 and MDA-MB-231) were isolated by sorting for $CD44^{+}CD24^{-/low}$ cells. The CSC populations of mouse TNBC cells (4T1) were isolated by sorting for $CD44^{+}CD24^{+}ALDH1^{+}$ cells.

(d) Bar graph showing the differential sensitivity to PBMC-mediated cancer cell killing between CSC and non-CSC populations of BT-549 cells in the presence or absence of PD-L1. BT-549 cells were incubated with activated PBMCs for 48 hours. After incubation, PBMC-mediated cancer cell killing on CSC and non-CSC populations was measured by flow cytometric analysis of cleaved caspase 3. Error bars represent s.d. (n = 3). *: $P < 0.05$; ns: non-significant, Student's t-test.

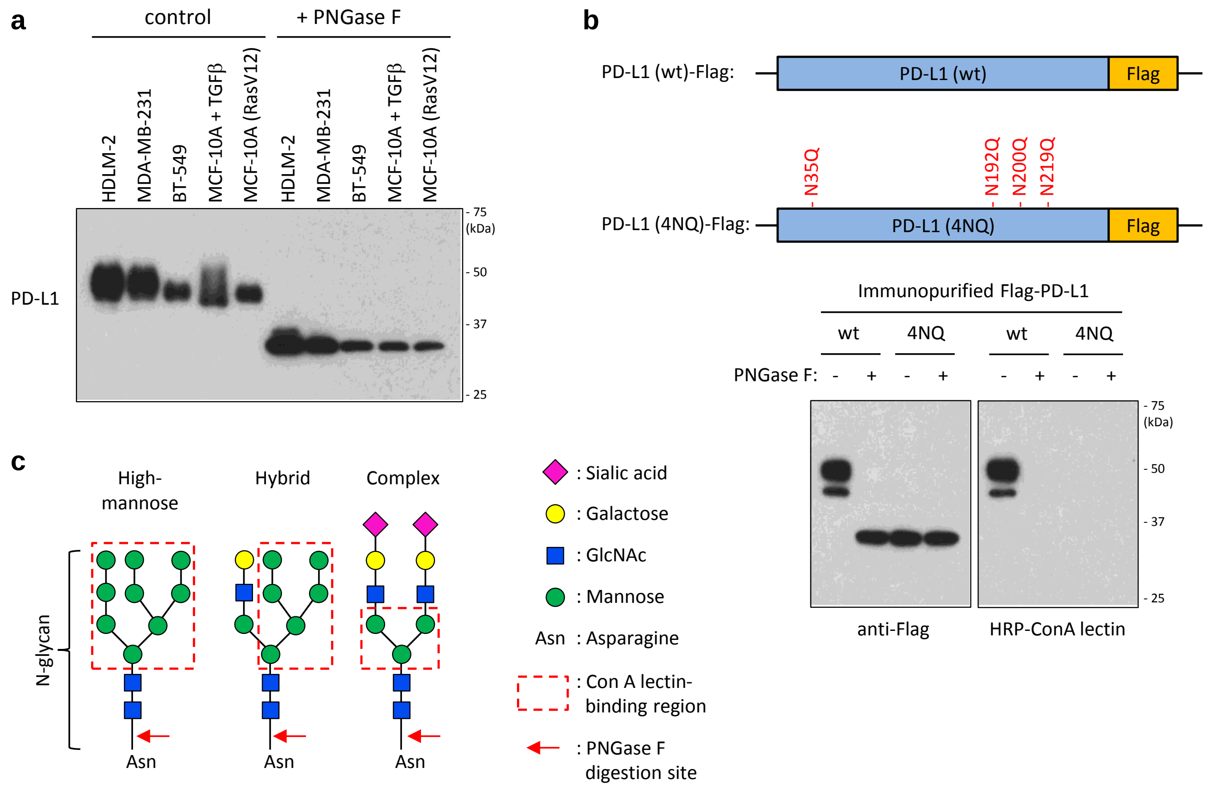

Supplementary Figure 2. PD-L1 is modified by N-glycosylation.

(a) Glycosylation pattern of PD-L1 protein in various cell lines. Cell lysates were treated with N-glycosidase F (PNGase F) to remove N-linked oligosaccharides and analyzed by western blotting.

(b) Top: Schematic illustrating wild-type (wt) PD-L1 and its glycosylation-site mutant (4NQ). The four glycosylation sites of PD-L1 are indicated. Bottom: Concanavalin A (ConA) lectin binding assay showing ConA lectin binding specificity to glycosylated PD-L1 (wt) and unglycosylated PD-L1 in which glycosylation was abolished by PNGase F or mutagenesis (4NQ).

(c) Schematic illustrating the three major types of N-glycan, including high-mannose, hybrid and complex glycans. ConA lectin-binding regions and PNGase F digestion sites are indicated.

a

NG site	Tryptic Peptide Sequence	Theoretical m/z of NxT	Theoretical m/z of DxT	N-glycan site occupancy (%) Epithelial cells	N-glycan site occupancy (%) Mesenchymal cells
35	K.DLYVVEYGSN^{35}MTIECK.F	968.9369	969.4289	10.6	93.3
192	K.LFN^{192}VTSTLR.I	525.8007	526.2927	28.8	99.5
200	R.IN^{200}TTTNEIFYCTFR.R	890.4221	890.9141	38.1	99.7
219	R.RLDPEEN^{219}HTAELVIPELPLAHPPNER.T	708.3646	708.6106	n/a	n/a

b

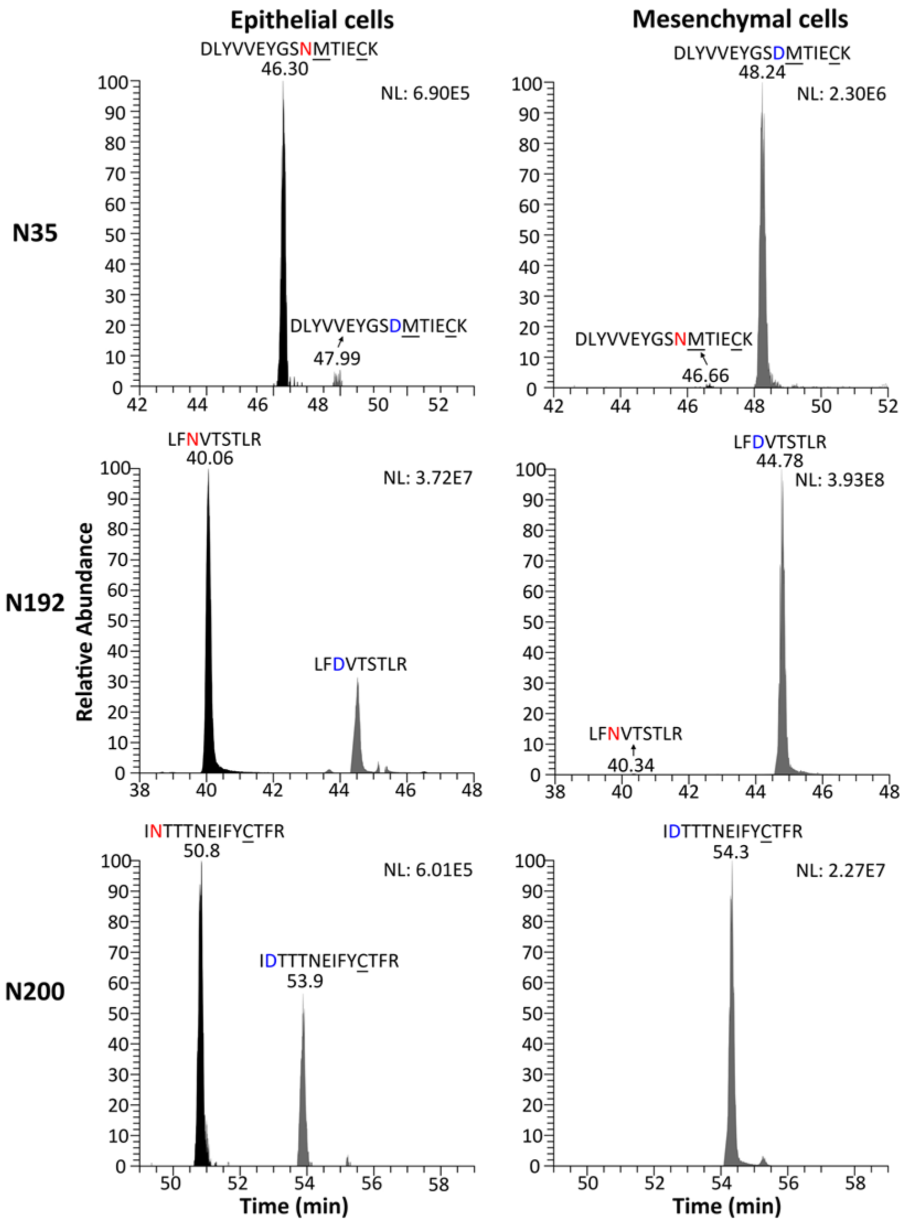

Supplementary Figure 3. Quantification of N-glycan site occupancy of each PD-L1 N-glycosylation site upon EMT.

(a) Table summarizing the N-glycan occupancy (%) of N35, N192 and N200 of PD-L1 purified from epithelial and mesenchymal cells.

(b) The extracted ion chromatograms for the PNGase F treated tryptic peptides corresponding to N35, N192 and N200 of PD-L1 purified from epithelial and mesenchymal cells.

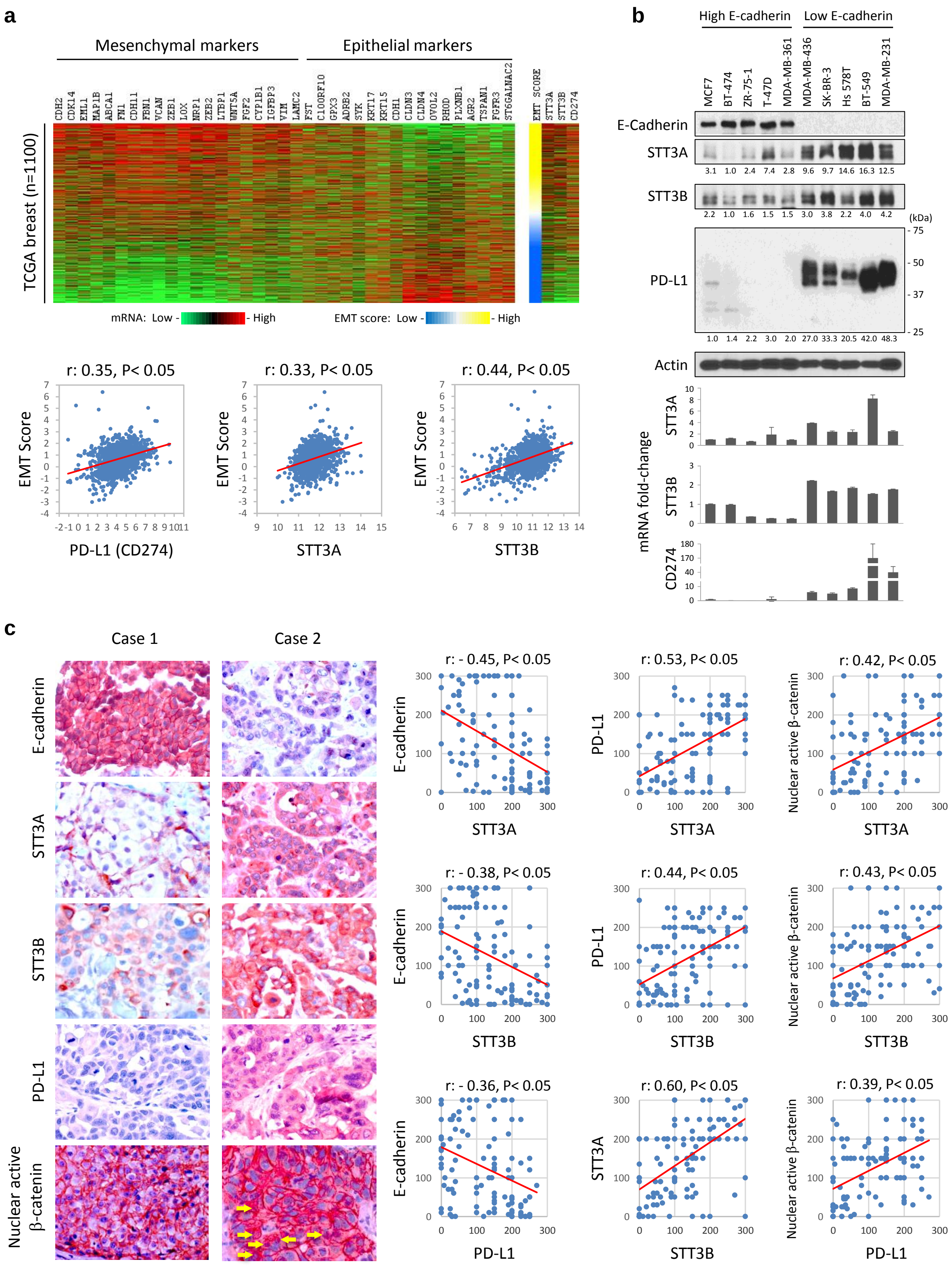
a
Mesenchymal markers
Epithelial markers
TCGA breast (n=1100)
mRNA: Low - - High
EMT score: Low - - High
r: 0.35, P< 0.05
r: 0.33, P< 0.05
r: 0.44, P< 0.05
EMT Score
PD-L1 (CD274)
STT3A
STT3B
b
High E-cadherin
Low E-cadherin
MCF7
BT-474
ZR-75-1
T-47D
MDA-MB-361
MDA-MB-436
SK-BR-3
Hs 578T
BT-549
MDA-MB-231
E-Cadherin
STT3A
STT3B
PD-L1
Actin
(kDa)
mRNA fold-change
CD274
c
Case 1
Case 2
E-cadherin
STT3A
STT3B
PD-L1
Nuclear active β-catenin
r: - 0.45, P< 0.05
r: 0.53, P< 0.05
r: 0.42, P< 0.05
r: - 0.38, P< 0.05
r: 0.44, P< 0.05
r: 0.43, P< 0.05
r: - 0.36, P< 0.05
r: 0.60, P< 0.05
r: 0.39, P< 0.05

Supplementary Figure 4. N-glycosyltransferases STT3A and STT3B are positively correlated with EMT.

(a) Pearson correlation analysis of EMT score with PD-L1 (CD274), STT3A and STT3B mRNA expressions in TCGA breast cancer dataset (n = 1100).

(b) Western blot and qRT-PCR analysis comparing STT3A, STT3B and PD-L1 protein (top) and mRNA (bottom) expression levels between high E-cadherin-expressing and low E-cadherin-expressing breast cancer cells. The signal intensity of STT3A, STT3B and PD-L1 blots were quantified and normalized relative to the lowest signal.

(c) Correlation analysis between epithelial marker E-cadherin, STT3A, STT3B, PD-L1 and nuclear non-phosphorylated (active) β-catenin at protein levels in breast cancer tissues (n = 129). Left: Representative images from immunohistochemistry staining of E-cadherin, STT3A, STT3B, PD-L1 and nuclear non-phosphorylated (active) β-catenin (indicated by arrows) in primary breast cancer tissues. Right: Pearson correlation analysis of immunohistochemistry staining results.

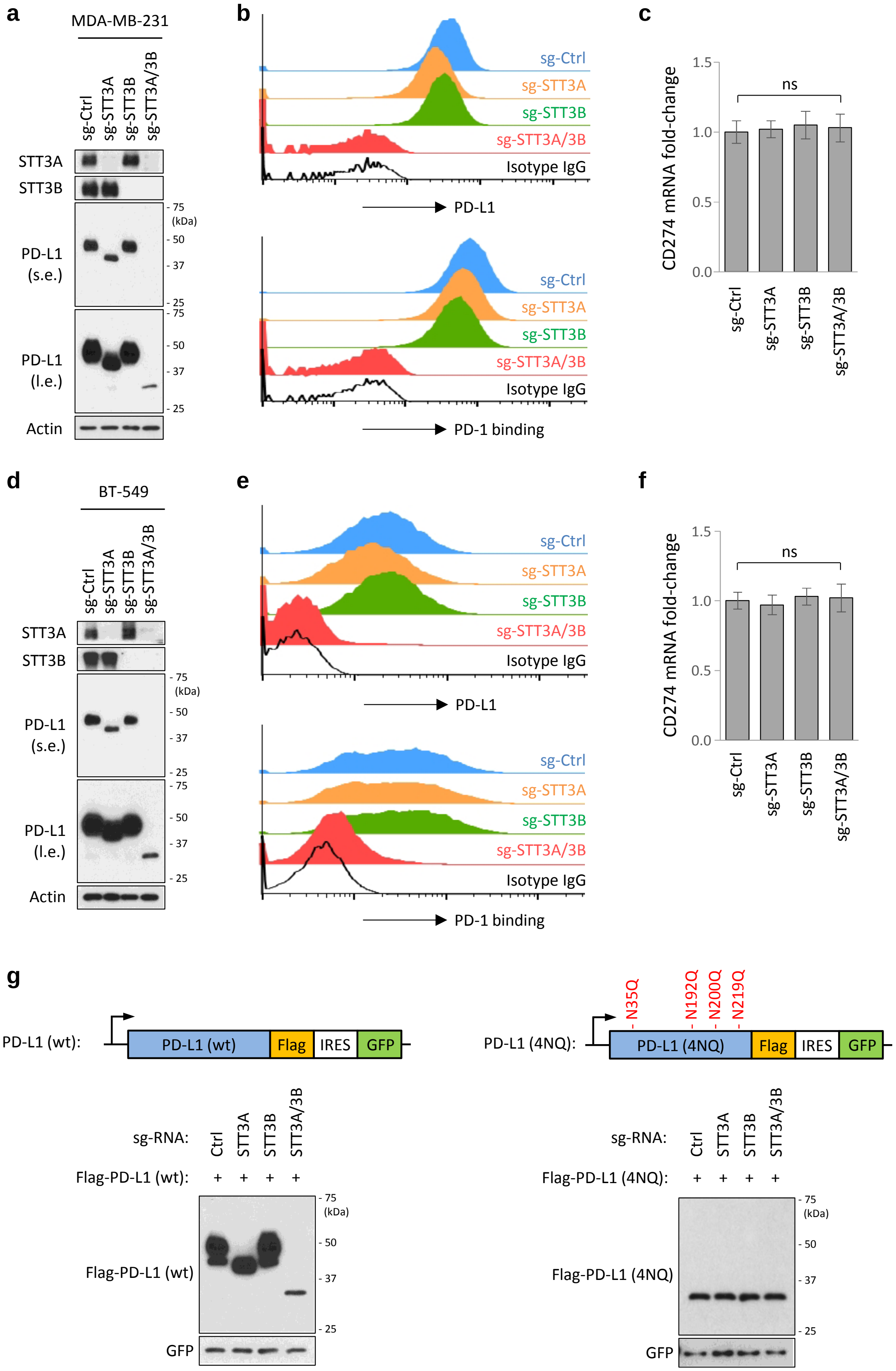
a
MDA-MB-231
sg-Ctrl
sg-STT3A
sg-STT3B
sg-STT3A/3B
STT3A
STT3B
PD-L1 (s.e.)
PD-L1 (l.e.)
Actin
75
(kDa)
50
37
25
b
sg-Ctrl
sg-STT3A
sg-STT3B
sg-STT3A/3B
Isotype IgG
PD-L1
PD-1 binding
c
CD274 mRNA fold-change
ns
1.5
1.0
0.5
0.0
d
BT-549
e
f
g
PD-L1 (wt):
PD-L1 (wt)
Flag
IRES
GFP
PD-L1 (4NQ):
PD-L1 (4NQ)
N35Q
N192Q
N200Q
N219Q
sg-RNA:
Ctrl
STT3A
STT3B
STT3A/3B
Flag-PD-L1 (wt):
Flag-PD-L1 (wt)
Flag-PD-L1 (4NQ):
Flag-PD-L1 (4NQ)
GFP

Supplementary Figure 5. Knockdown of both STT3 isoforms suppresses PD-L1 protein expression without significant reduction of PD-L1 mRNA levels in mesenchymal-like breast cancer cells.

(a, d) Effect of STT3 isoforms knockdown on the expression and molecular weight of endogenous PD-L1 protein in MDA-MB-231 (**a**) and BT-549 (**d**) cells.

(b, e) Flow cytometric analysis of the effect of STT3 isoforms knockdown on the cell surface PD-L1 expression and PD-1 binding ability of MDA-MB-231 (**b**) and BT-549 (**e**) cells.

(c, f) Influence of STT3 isoforms knockdown on PD-L1 (CD274) mRNA levels in MDA-MB-231 (**c**) and BT-549 (**f**) cells.

(g) Top: Schematic illustrating co-expression constructs of PD-L1 (wt or 4NQ) and green fluorescent protein (GFP) used to assay the protein expression status of PD-L1. GFP was used as an internal control for transfection efficiency and gene expression. IRES: internal ribosome entry site. Bottom: Effect of STT3 isoforms knockdown on the protein expression amounts of PD-L1 (wt) or (4NQ) protein in BT-549 cells.

Error bars represent s.d. ($n = 3$). *: $P < 0.05$; ns: non-significant, Student's t-test.

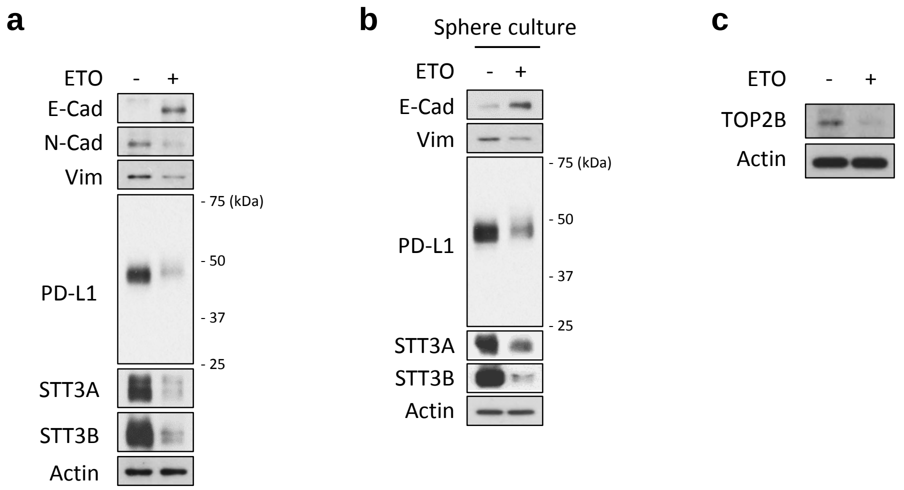

Supplementary Figure 6. Etoposide induces MET and downregulation of STT3 and PD-L1 in MDA-MB-231 cells.

(a) Effect of etoposide (5 μg/ml, 4 days) on the expression of EMT markers, STT3 isoforms and PD-L1 in the general cell population of MDA-MB-231 cells.

(b) Western blot analysis of EMT markers, STT3 isoforms and PD-L1 of tumorspheres derived from etoposide-treated MDA-MB-231 cells.

(c) Effect of etoposide on TOP2B expression in the general cell population of MDA-MB-231 cells.

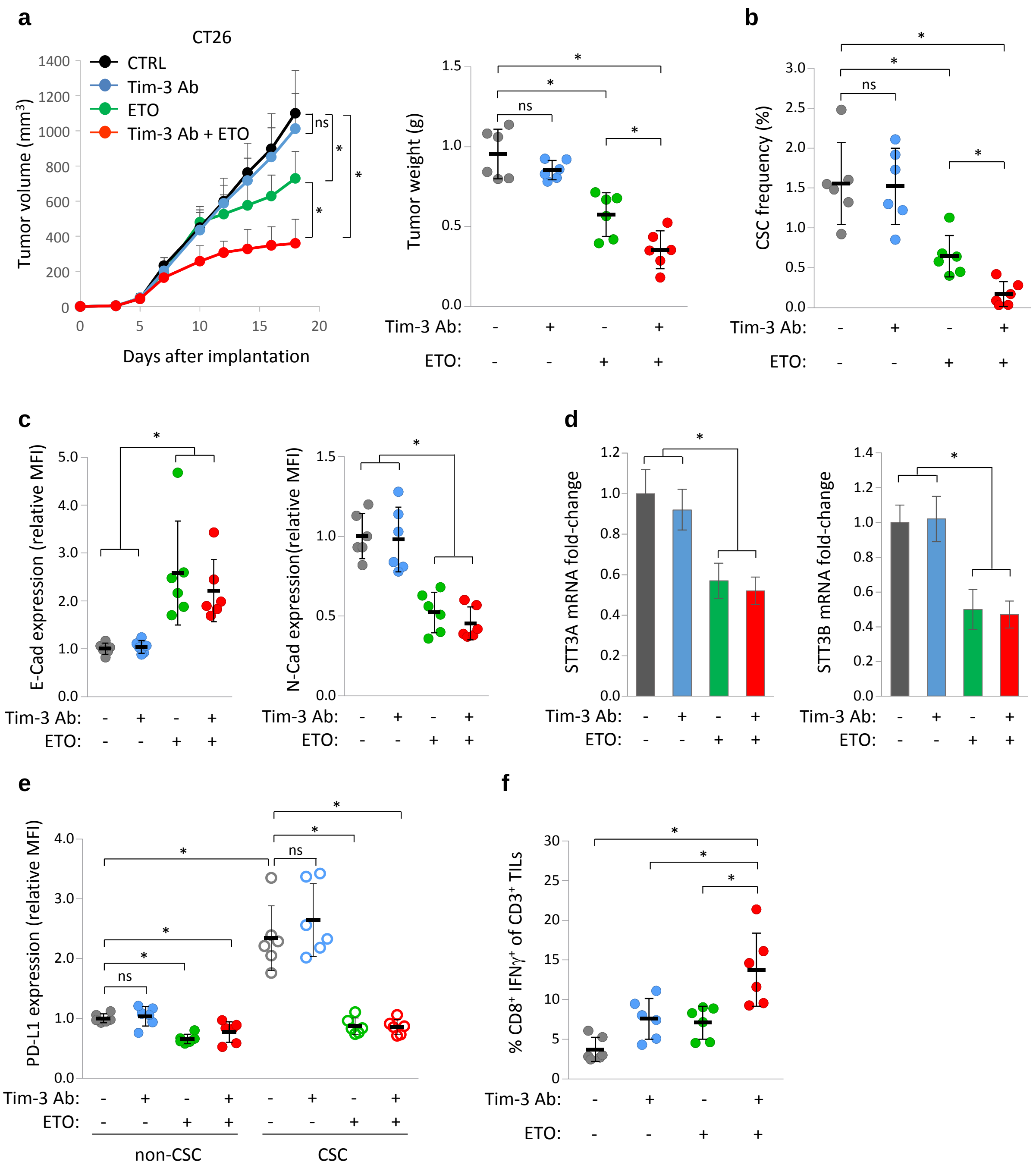

a
CT26
CTRL
Tim-3 Ab
ETO
Tim-3 Ab + ETO
Tumor volume (mm³)
Days after implantation
Tumor weight (g)
Tim-3 Ab:
ETO:
ns
*
b
CSC frequency (%)
c
E-Cad expression (relative MFI)
N-Cad expression(relative MFI)
d
STT3A mRNA fold-change
STT3B mRNA fold-change
e
PD-L1 expression (relative MFI)
non-CSC
CSC
f
% CD8+ IFNγ+ of CD3+ TILs

Supplementary Figure 7. Etoposide synergizes with Tim-3 blockade therapy in CT26 colorectal tumor model.

(a) Tumor growth of CT26 cells in BALB/c mice treated with Tim-3 antibody (Tim-3 Ab) and/or etoposide (ETO). Tumor size was measured at the indicated time points and tumor weight was measured at the endpoint (n = 6 mice per group).

(b) Efficacy of indicated treatments on the CSC ($CD44^+CD133^+ALDH1^+$ populations) frequency of CT26 tumors.

(c) Flow cytometric analysis comparing the effect of indicated treatments on the expression of E-cadherin (E-Cad, epithelial marker) and N-cadherin (N-Cad, mesenchymal marker) in the entire cancer cell population of CT26 tumors.

(d) qRT-PCR analysis of the influence of indicated treatments on STT3 isoforms expression in the entire cancer cell population of CT26 tumors.

(e) Effect of indicated treatments on PD-L1 expression in the CSC and non-CSC populations of CT26 tumors.

(f) Intracellular cytokine staining of $CD8^+$ $IFN\gamma^+$ cells in the $CD3^+$ T cell populations from isolated tumor-infiltrating lymphocytes to analyze the impacts of indicated treatments on tumor-infiltrating cytotoxic T cell activity.

Error bars represent s.d. (n = 6). $^*P < 0.05$; ns: non-significant, Student's t-test.

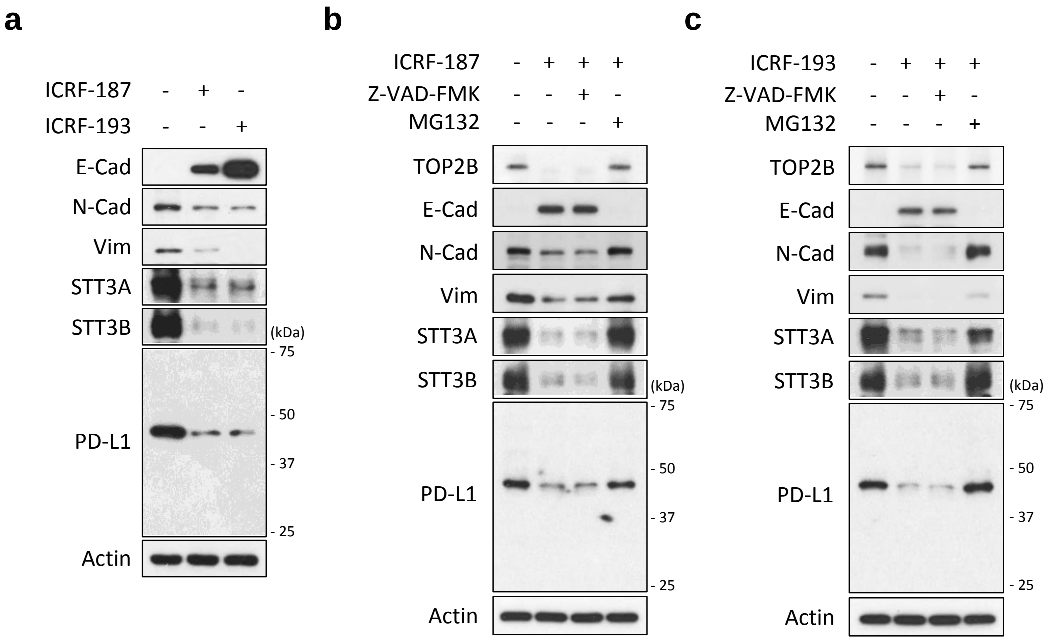

Supplementary Figure 8. Proteasome inhibitor MG132 blocks TOP2 inhibitor-induced MET and downregulation of TOP2B, STT3 isoforms and PD-L1.

(a) Effect of TOP2 inhibitors ICRF-187 and -193 (5 μM, 24 hr) on the expression of EMT markers, STT3 isoforms and PD-L1 in 4T1 cells.

(b, c) Effect of proteasome inhibitor (MG132) and caspase inhibitor (Z-VAD-FMK) on TOP2 inhibitor-induced TOP2B degradation, MET and downregulation of STT3 and PD-L1.

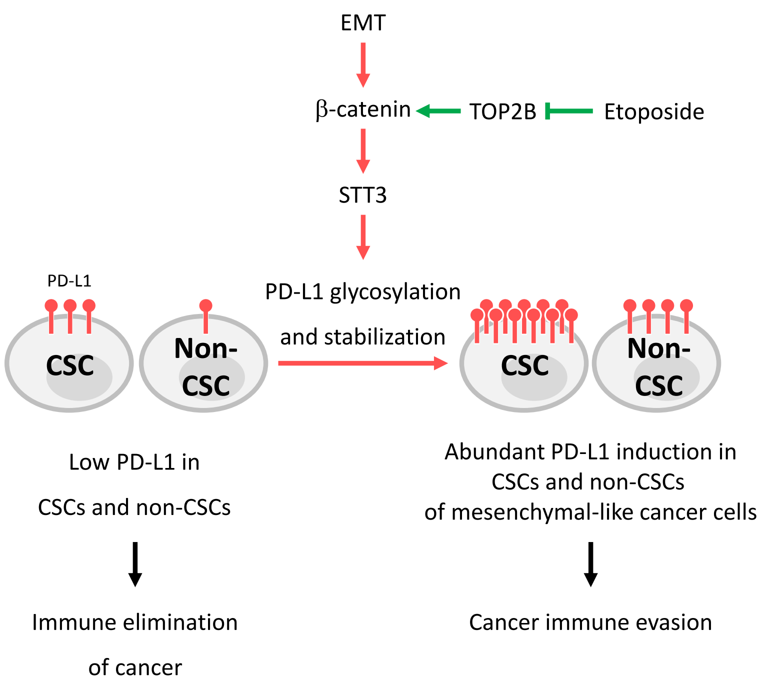

Supplementary Figure 9. Schematic illustrating the regulation of PD-L1 in CSCs and non-CSCs by the EMT/β-catenin/STT3/PD-L1 axis and etoposide. EMT induces N-glycosyltransferase STT3 through β-catenin. STT3 positively regulates PD-L1 glycosylation and stabilization, leading to PD-L1 induction in CSCs and non-CSCs. High PD-L1 expression contributes to cancer immune evasion. Etoposide reduces nuclear β-catenin through TOP2B degradation and inhibits the EMT-β-catenin-STT3-PD-L1 signaling axis, resulting in sensitization of both CSCs and non-CSCs to immune elimination.

Fig. 1a

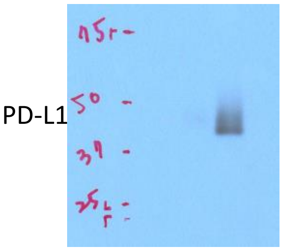

Fig. 1c

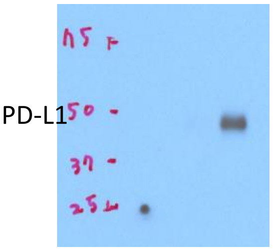

Fig. 1e

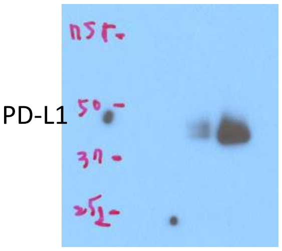

Fig. 1f

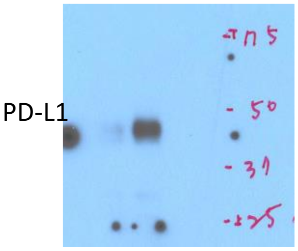

Fig. 2a

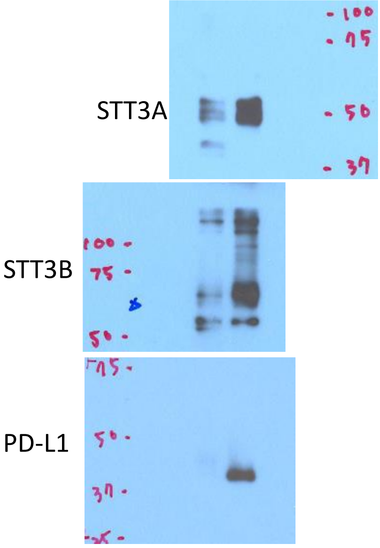

Fig. 2d

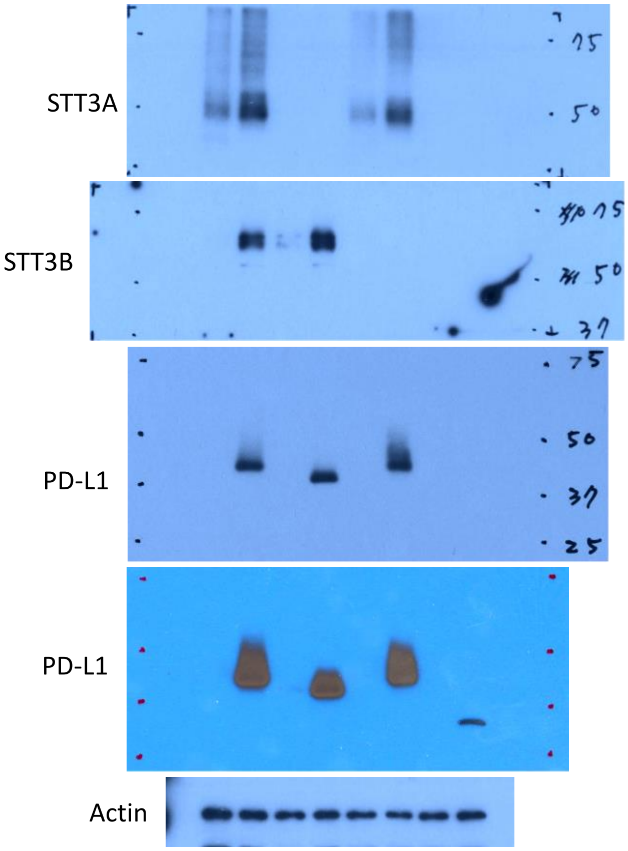

Fig. 2c

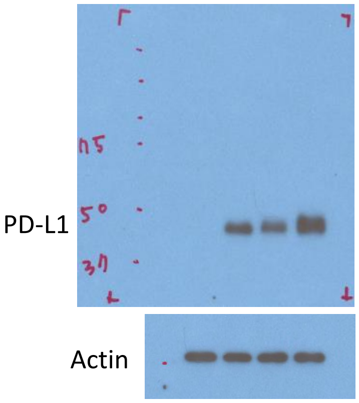

Fig. 2h

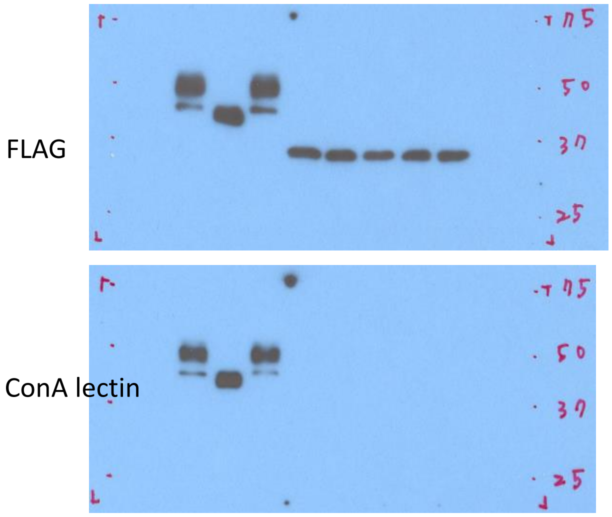

Fig. 2i

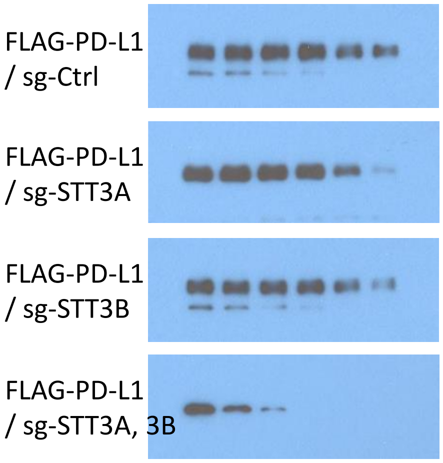

Fig. 2j

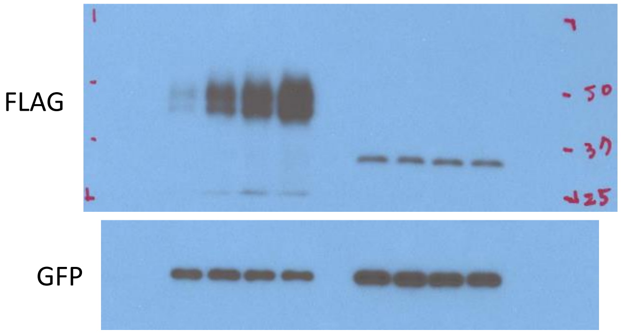

Fig. 3a

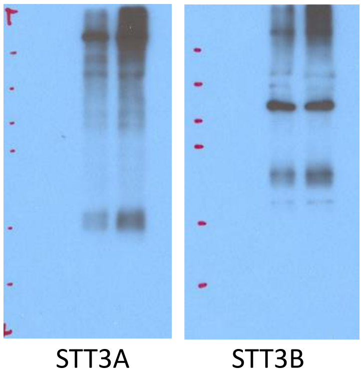

Fig. 3b

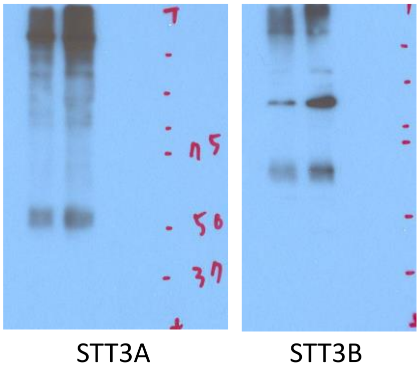

Fig. 3c

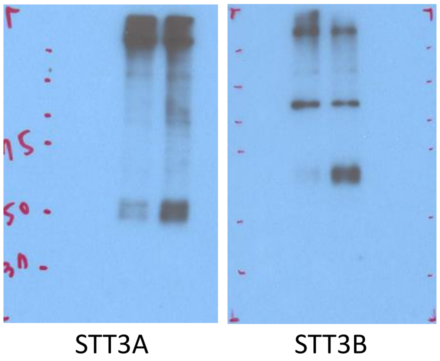

Fig. 4d

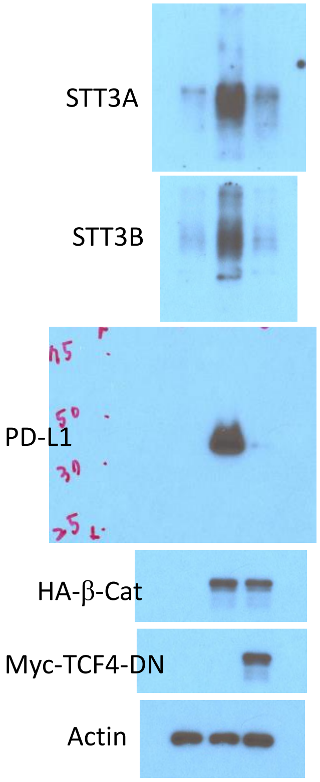

Fig. 4e

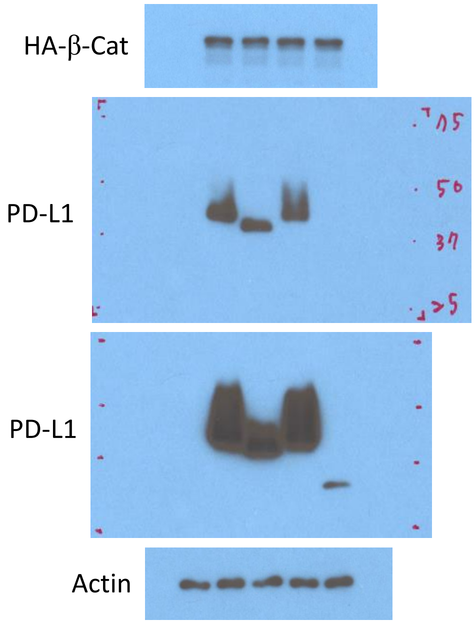

Fig. 4f

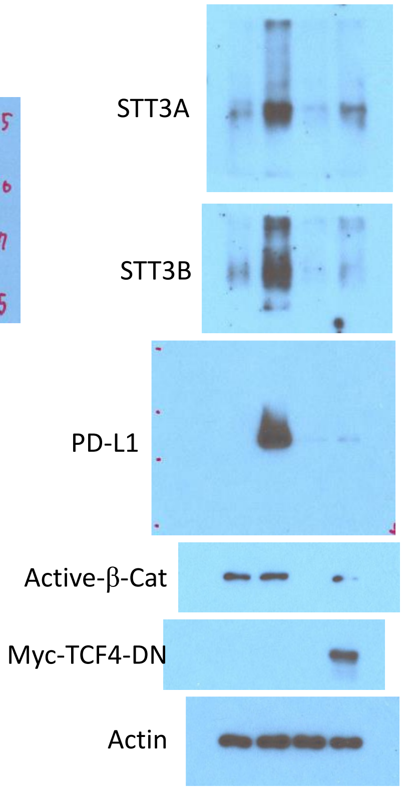

Fig. 4g

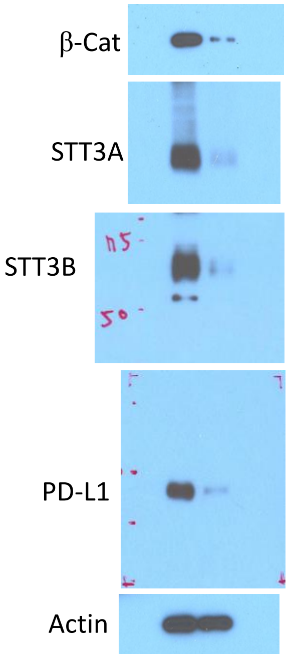

Fig. 5a

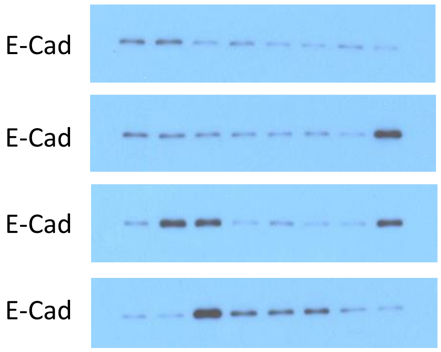

Fig. 6a

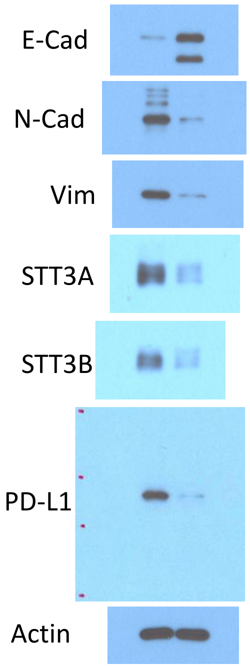

Fig. 6b

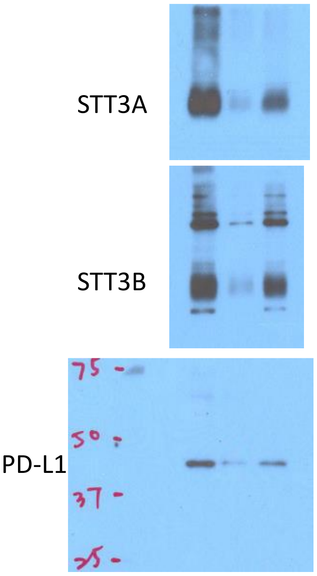

Fig. 6e

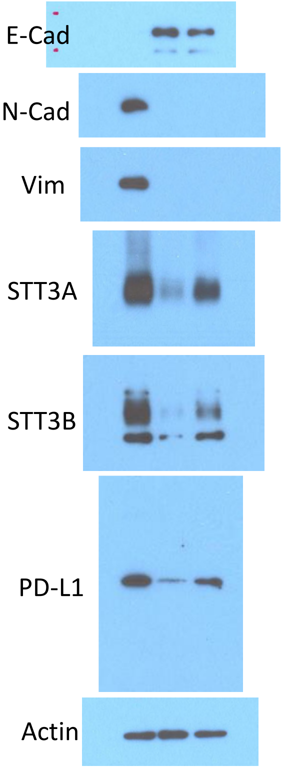

Fig. 8a

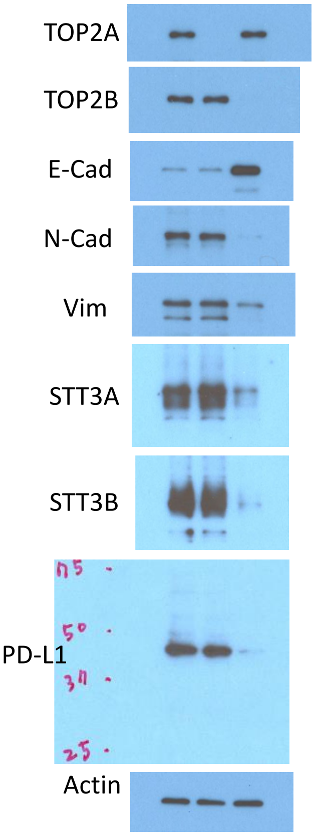

Fig. 8c

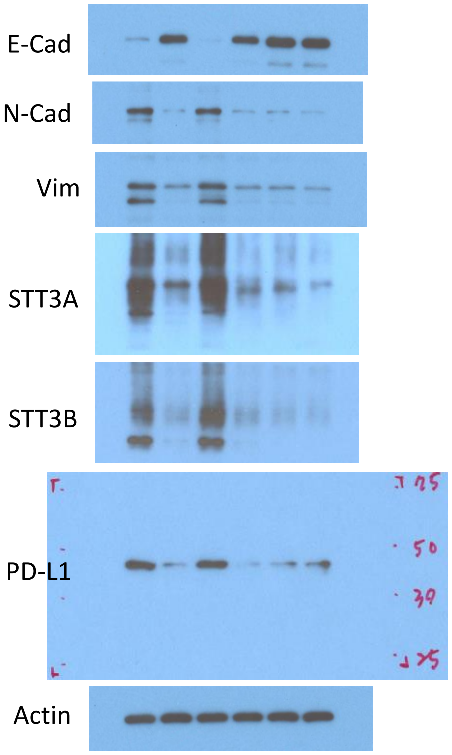

Fig. 8d

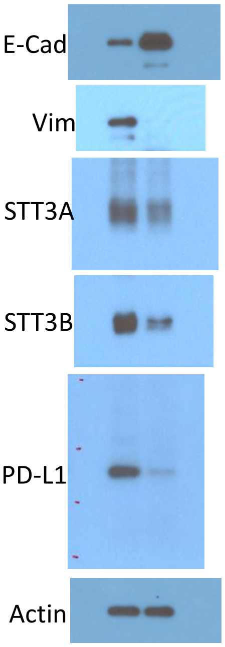

Fig. 8e

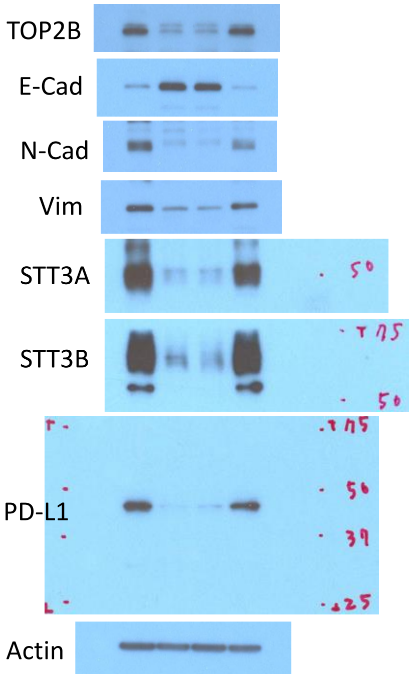

Fig. 8f

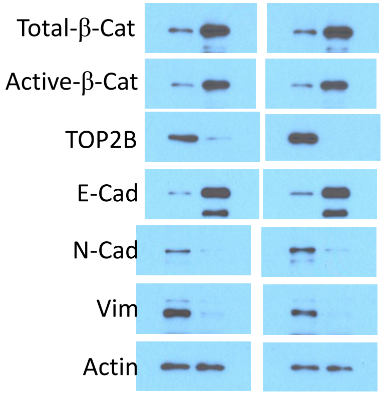

Fig. 8g

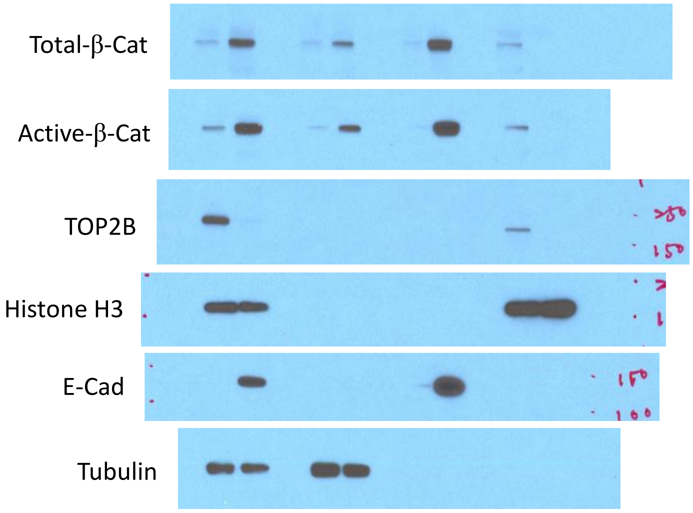

Fig. 8h

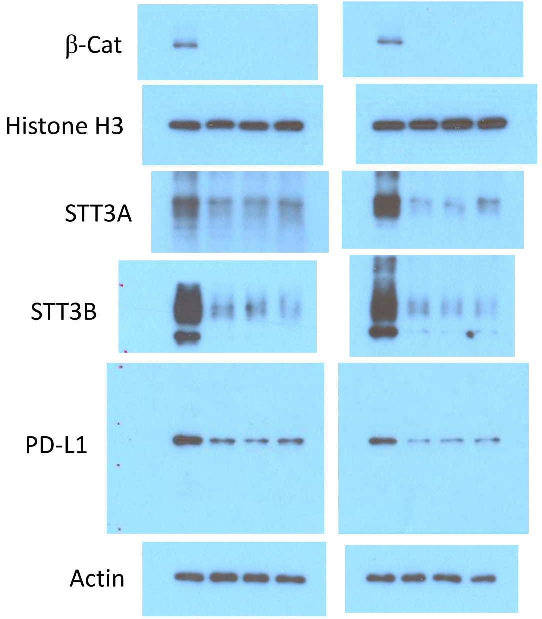

Fig. 8i

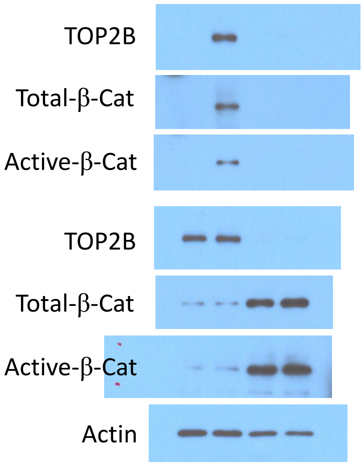

Supplementary Figure 10. Uncropped scans of the Western blots shown in the indicated figures.

Supplementary Table 1, related to Figure 5. List of the chemical compound concentrations used in Figure 5.

Chemical compound	Concentration for 24-h treatment (μM)	Concentration for 96-h treatment (μM)
Chlorambucil	25	
Cyclophosphamide	50	
Carmustine	15	
Busulfan	5	
Dacarbazine	15	
Thiotepa	5	
Cisplatin	5	
Carboplatin	20	
5-Fluorouracil	1	
6-Mercaptopurine	5	
Gemcitabine	0.01	
Methotrexate	0.5	
Pentostatin	2	
Daunorubicin	1	0.02
Doxorubicin	2	0.05
Epirubicin	1	0.02
Actinomycin-D	0.1	
Mitomycin-C	2	
Topotecan	1	
Irinotecan	5	
Etoposide	10	0.2
Teniposide	15	
Mitoxantrone	2	0.05
Paclitaxel	1	0.02
Docetaxel	2	
Vincristine	1	

Supplementary Table 2. Oligonucleotide sequences used throughout this study.

Gene knockdown

sgRNA or siRNA (vendor, catalogue number)	Target sequence (listed 5' to 3')
Human STT3A sgRNA (Santa Cruz, sc-405155)	ACAGACATTCCGAATGTCGA<br>AAGGTGGTACGTGACGATGG<br>CTCGGTCATCAAACCAGTTA
Human STT3B sgRNA (Santa Cruz, sc-404481)	GATGTAAGGCCGCTAAAAGT<br>CCAGCGGTTATCATCAACCC<br>TACAGCAAAAGAGTCTACAT
Human β-catenin siRNA (Sigma, EHU139421)	GCCGGCTATTGTAGAAGCTGGTGGA<br>ATGCAAGCTTTAGGACTTCACCTGA<br>CAGATCCAAGTCAACGTCTTGTTCA<br>GAACTGTCTTTGGACTCTCAGGAAT<br>CTTTCAGATGCTGCAACTAAACAGG<br>AAGGGATGGAAGGTCTCCTTGGGAC<br>TCTTGTTCAGCTTCTGGGTTCAGAT<br>GATATAAATGTGGTCACCTGTGCAG<br>CTGGAATTCTTTCTAACCTCACTTG<br>CAATAATTATAAGAACAAGATGATG<br>GTCTGCCAAGTGGGTGGTATAGAGG<br>CTCTTGTGCGTACTGTCCTTCGGGC<br>TGGTGACAGGGAAGACATCACTGAG<br>CCTGCCATCTGTGCTCTTCGTCATC<br>TGACCAGCCGACACCAAGAAGCAGA<br>GATGGCCCAGAATGCAGTTCGCCTT<br>CACTATGGACTACCAGTTGTGGTTA<br>AGCTCTTACACCCACCATCCCACT
Mouse β-catenin siRNA (Sigma, EMU047621)	AGGGTGGGAATGGTTTTAGGCCTGT<br>TTGTAAATCTGCCACCAAACAGATA<br>CATACCTTGGAAGGAGATGTTCATG<br>TGTGGAAGTTTCTCACGTTGATGTT<br>TTTGCCACAGCTTTTGCAGCGTTAT<br>ACTCAGATGAGTAACATTTGCTGTT<br>TTCAACATTAATAGCAGCCTTTCTC<br>TCTATACAGCTGTAGTGTCTGAACG<br>TGCATTGTGATTGGCCTGTAGAGTT<br>GCTGAGAGGGCTCGAGGGGTGGGCT<br>GGTATCTCAGAAAGTGCCTGACACA<br>CTAACCAAGCTGAGTTTCCTATGGG<br>AACAGTCGAAGTACGCTTTTTGTTC<br>TGGTCCTTTTTGGTCGAGGAGTAAC<br>AATACAAATGGATTTGGGGAGTGAC<br>TCACGCAGTGAAGAATGCACACGAA<br>TGGATCACAAG

Mouse TOP2A sgRNA (Santa Cruz, sc-423469)	CTCATCGTCGTCATAGTTAC<br>CTCCGCCCAGATACCTACAT<br>TGGGTTTACGATGAAGATGT
Mouse TOP2B sgRNA (Santa Cruz, sc-423470)	CTTCGTCCTGATACATACAT<br>ACTGATCCAATGTATGTATC<br>TCTACTTTGTGTTCTACTAC

qRT-PCR

Target gene	Primer sequence (listed 5' to 3')
Human CD274	TGCCGACTACAAGCGAATTACTG<br>CTGCTTGTCCAGATGACTTCGG
Mouse CD274	TGCGGACTACAAGCGAATCACG<br>CTCAGCTTCTGGATAACCCTCG
Human STT3A	GAAGCAACAGGATTCCACCTACC<br>CAATGGACGGAGAAGAGTAGGC
Mouse STT3A	GCAACCTGTCTGATGCTCGGAT<br>ATGTGGACAGCACCTGGGAAAC
Human STT3B	GCAGGTGCTGTGTTCCTTAGTG<br>GTCGTAGGTTGATGCTCAGACAC
Mouse STT3B	CCTACTCCAGTCCAAGTGTGGT<br>GCCAGCAATCTGATAGCCGTAG
Mouse CDH1	GGTCATCAGTGTGCTCACCTCT<br>GCTGTTGTGCTCAAGCCTTCAC
Mouse VIM	CGGAAAGTGGAATCCTTGCAGG<br>AGCAGTGAGGTCAGGCTTGGAA
