# Supplementary material for: STT3-dependent PD-L1 accumulation on cancer stem cells promotes immune evasion
Source: Nat Commun. 2018 May 15;9:1908. doi: 10.1038/s41467-018-04313-6 (PMC5954021; doi:10.1038/s41467-018-04313-6)
Supplement: Supplementary file 3 — Description of Additional Supplementary Files [file 41467_2018_4313_MOESM3_ESM.pdf]

Description of Additional Supplementary Files

File Name: Supplementary Data 1
Description: EMT scores calculated based on mRNA expression signatures of 37 core EMT-related genes, including 20 mesenchymal genes and 17 epithelial genes.
